# Phylogeography of the Black Kite (*Milvus migrans*) in Punjab Wetlands: Assessing genetic connectivity and lineage admixture at a migratory crossroads

**DOI:** 10.1371/journal.pone.0351642

**Published:** 2026-06-29

**Authors:** Muhammad Zeshan Haider, Gulnaz Afzal, Hafiz Ishfaq Ahmad, Javed Hussain, Iram Qadeer, Shakeel Ahmed, Laila A. AL-Essa, Nisar Ahmad Koka, Aiman A. Alsaegh, Yehia Hazzazi

**Affiliations:** 1 Department of Zoology, The Islamia University of Bahawalpur, Bahawalpur, Pakistan; 2 Department of Animal Breeding and Genetics, Faculty of Veterinary and Animal Sciences, The Islamia University of Bahawalpur, Bahawalpur, Pakistan; 3 Department of Zoology, The Govt. Sadiq College Women University, Bahawalpur, Pakistan; 4 Environment Agency – Abu Dhabi, Abu Dhabi, United Arab Emirates; 5 Department of Mathematical Sciences, College of Science, Princess Nourah bint Abdulrahman University, Riyadh, Saudi Arabia; 6 English Language Center, Vice-Presidency of Academic and Educational Affairs, King Khalid University, Abha, Kingdom of Saudi Arabia; 7 Department of Clinical Laboratory Sciences, Faculty of Applied Medical Sciences, Umm Al-Qura University, Makkah, Saudi Arabia; 8 Department of Biology, College of Science, Jazan University, Jazan, Kingdom of Saudi Arabia; Imam Abdulrahman Bin Faisal University, SAUDI ARABIA

## Abstract

The *Milvus* genus presents a taxonomic challenge due to the complex delineation of its evolutionary units. Research on the potentially divergent mitochondrial lineages within *Milvus migrans* in Pakistan remains sparse, leaving the regional phylogeny largely uncharacterized. This study utilized partial *COI* gene sequences to evaluate genetic relationships and identify the presence of divergent lineages within Pakistan’s *M. migrans* populations. Samples collected from three Punjab wetlands (Chashma Barrage, Taunsa Barrage, and Patisar Lake) were integrated with global GenBank reference sequences to evaluate maternal diversity and reconstruct demographic histories. Maternal genetic distances among regional conspecifics were remarkably low, ranging from 0.00% to 1.00%. However, comparisons across more divergent mitochondrial lineages revealed distances reaching 2.00%, particularly between West African and European isolates; notably, these intraspecific values remained distinctly lower than the significant divergence observed with outgroup taxa. Neutrality tests indicated signatures consistent with population expansion or purifying selection. A hierarchical AMOVA confirmed strong continental-scale genetic structuring (65.39% of total variation) contrasting with high gene flow within Punjab’s panmictic local populations. Principal Coordinates Analysis (PCoA) revealed lineage-driven clustering, supporting a homogeneous admixture of three mitochondrial lineages within the Indus Flyway. These results collectively reveal a significant intersection of divergent lineages within Pakistan’s *M. migrans* populations, highlighting the Punjab region as a critical zone of genetic admixture. While the data indicate high maternal connectivity and extensive haplotype sharing with distant global lineages, they also hint at complex evolutionary histories. These findings underscore the ecological importance of Punjab wetlands as a migratory hub, though the hypothesis of cryptic diversity remains tentative and requires future validation via multi-locus nuclear genomic markers.

## Introduction

The Black Kite (*Milvus migrans*), belonging to the family Accipitridae, is confined to the Old World distributed across Eurasia, Africa, and Australia [1,2]. In Pakistan, Black Kites are common across wetlands and urban centers, where they depend heavily on human waste and carrion [3]. Despite its broad distribution, *Milvus* lineages face escalating threats from habitat loss, pollution, agrochemical poisoning [4], and direct persecution [5,6]. Such pressures necessitate a robust understanding of population genetics to inform effective management [7]. While the European *M. migrans* breeding population is well-studied, representing about 10% of the global total, genetic and demographic data for most of its vast Asian and African mitochondrial lineages remain unknown, posing a significant challenge to global conservation efforts [8].

Phylogeography, the study of spatial patterns in genetic lineages, provides critical insights into evolutionary history and population connectivity across broad ranges [9]. The taxonomy of *Milvus migrans* is complex, with five to seven recognized subspecies, including *M. m. migrans* (European Black Kite), *M. m. lineatus* (Black-eared Kite), and *M. m. govinda* (Pariah Kite) [2,10]. These subspecies exhibit overlapping distributions and intergradation zones where hybridization occurs [11], notably between *migrans* and *lineatus* across Western Siberia and Eastern Europe [12]. Evidence suggests that this contact zone may extend toward the northwestern Himalayas, encompassing Afghanistan and Pakistan, potentially involving all three Eurasian subspecies [11]. Phenotypic similarities between migratory and sedentary groups further complicate the identification of lineages, highlighting the need for molecular resolution [7].

In this context, molecular tools, particularly DNA barcoding, have emerged as indispensable for modern biodiversity research and species identification [13]. This powerful technique involves the rapid and cost-effective analysis of specific mitochondrial DNA regions, notably the cytochrome c oxidase subunit I (*COI*) gene, to identify species and infer evolutionary relationships [14]. As a protein-coding mitochondrial marker, the *COI* gene is characterized by rapid evolution and a lack of recombination. These attributes make it ideal for resolving genetic structure, identifying divergent lineages, and reconstructing phylogenetic histories in avian species [15,16].

Pakistan is situated at the confluence of several major avian flyways, most notably serving as the axis for the ‘Indus Flyway’ (International Migratory Bird Route Number 4), also known as the Green Route [17,18]. Stretching from the Karakoram ranges down to the Indus Delta, this corridor is a critical biological artery for over 400 migratory bird species that undertake an exhaustive 4,500 km journey from Siberia and Central Asia [19,20]. The Punjab wetlands situated centrally along the Indus basin are vital migratory bird areas, representing a critical ecological hub for both resident and migratory *Milvus* species and sub-species, yet their genetic composition remains unexplored [21,22]. Despite the high ecological stakes, critical knowledge gaps regarding the genetic diversity and population structure of raptors along this route hinder evidence-based conservation [23]. This study, therefore, focused on amplifying and sequencing the mitochondrial *COI* gene from *M. migrans* specimens collected across Chashma Barrage, Taunsa Barrage, and Patisar Lake wetlands of Punjab, Pakistan. We integrated our Punjab sequences with a global GenBank dataset to contextualize regional findings within a broader phylogenetic framework. This comparative approach facilitates a deeper analysis of lineage admixture and genetic connectivity at both regional and global scales. By examining local genetic diversity, phylogenetic relationships, and the dynamics of lineage admixture, we aim to elucidate the degree of genetic connectivity characterizing this critical migratory crossroads. This study serves as a foundational mitochondrial assessment, providing a baseline for future genomic inquiries into the potential for cryptic variation within the region.

## Materials and methods

### Ethical considerations

This study was conducted in rigorous adherence to the ethical protocols for animal research established by the Institutional Bio-Safety Committee (IBSC) and the Department of Zoology (Approval No. 1112/AS&R), The Islamia University of Bahawalpur, Pakistan. Official sanction was granted by the Institutional Animal Ethics Committee (IAEC) via letter No. 454/ORIC, dated December 19, 2024. All research procedures were executed and reported in accordance with the ARRIVE guidelines. To prioritize animal welfare, field procedures were limited to non-invasive handling and blood collection; no protocols involving anesthesia, euthanasia, or animal sacrifice were employed, and no instances of individual animal suffering were observed during the investigation.

### Study area

This study was conducted in three prominent wetlands in the Punjab province of Pakistan: Chashma Barrage (32°25’00.0"N, 71°22’00.0"E), Taunsa Barrage (30°30’46.0"N, 70°50’57.0"E), and Patisar Lake (29°20’41.2″N, 71°56’21.0″E). These locations are crucial stopover places along the Indus Flyway (Green Route), attracting both local and migratory avifauna, including *M. migrans* populations from Siberia, Russia, and northern regions [17,22]. Chashma Barrage (Site 1) designated wetland encompasses 33,082 hectares. The semi-arid climate, characterized by hot summers (up to 41°C in June) and cold winters (down to 4.5°C in January), provides a diverse environment for wintering migratory birds [24]. Taunsa Barrage (Site 2) is recognized as an Important Bird Area and a key component of the Indus Flyway. Its rich biodiversity includes globally threatened species and new records documented in our previous research [21]. Patisar Lake (Site 3) is located within Lal Suhanra National Park, with significant supporting bird species [24].

### Black Kite capture and blood collection

During fieldwork *Milvus migrans* were captured using the bal-chatri method, a widely recognized raptor capture technique by Berger and Mueller [25]. This trap, which consists of a wire cage adorned with monofilament nooses and containing animal flesh as a lure, is highly effective. The trap size and shape were adjusted according to the target species. A total of 18 *M. migrans* were captured (6 from each site) across three wetlands; Chashma Barrage, Taunsa Barrage, & Patisar Lake in Punjab, Pakistan. Each specimen was assigned a unique abbreviation for individual identification and locality tracking. Blood samples were collected in sterilized vacutainer tubes utilizing EDTA anticoagulant and stored in an ice box before DNA extraction. Following sample collection, all Black Kites were immediately released back into their natural habitat.

### DNA extraction

Genomic DNA was extracted from the collected blood samples using the Qiagen DNeasy Blood & Tissue Kit, following the standard spin-column protocol for total DNA purification from animal blood [26]. This protocol is optimized for samples containing nucleated erythrocytes, characteristic of avian species. Prior to extraction, blood samples were carefully thawed if previously frozen, and the manufacturer recommended protocol was strictly followed under sterile conditions. For each sample, 20 µl of Proteinase K was added to a 1.5 ml microcentrifuge tube containing 5–10 µl of anticoagulated blood. The volume was adjusted to 220 µl with phosphate-buffered saline (PBS). In some cases, 4 µl of RNase A (100 mg/ml) was added to digest RNA contamination, ensuring high-quality DNA isolation. The mixture was vortexed thoroughly for homogenization before adding 200 µl of Buffer AL. The solution was then incubated at 56°C for 10 minutes for lysis and protein degradation. Subsequently, 200 µl of ethanol (96–100%) was added to the lysate to promote DNA binding to the spin column silica membrane. The lysate was transferred to a DNeasy Mini spin column placed in a 2 ml collection tube and centrifuged using a Thermo Scientific™ Pico™ 21 Microcentrifuge at 6000 x g for 1 minute. The column underwent a wash sequence with Buffer AW1 followed by Buffer AW2, with flow-through discarded after centrifugation to purify membrane-bound DNA. A final wash was performed before a 3-minute centrifugation at 14,000 x g to dry the membrane and remove residual ethanol. DNA was eluted by applying 200 µl of Buffer AE directly to the membrane, incubating for one minute at room temperature, and then centrifuging at 6000 x g for one minute. The elution step was repeated to maximize DNA yield.

### PCR amplification and gel electrophoresis

The mitochondrial DNA cytochrome c oxidase subunit I (*COI*) gene was selected as a genetic marker due to its widespread use in avian genetic studies and its proven reliability for species identification and preliminary population structure analysis [11]. DNA amplification of the *COI* gene segment was performed using polymerase chain reaction (PCR) with the following primers: the forward primer 5’ GGTCAACAAATCATAAAGATATTGG 3’ and the reverse primer 5’ TAAACTTCAGGGTGACCAAAAAATCA 3’ [27]. PCR amplification was carried out in a thermal cycler (25 μl volume) with the following cycling conditions: initial denaturation at 95°C for 5 minutes, followed by 35 cycles of denaturation at 94°C for 45 seconds, annealing at 55°C for 35 seconds, and extension at 72°C for 45 seconds. A final extension step at 72°C for 5 minutes was included to ensure complete amplification of the target DNA segment. The PCR products were held at 10°C until further processing. The PCR products, approximately 700 bp in size, were separated by 1.5% agarose gel electrophoresis. The resulting DNA fragments were visualized using UV transillumination to confirm successful amplification (S1 Fig). PCR products from 18 samples selected randomly from all sampling sites were sent to the Celemics BTSeq™ Institute for sequencing performed by 1st BASE Services, Singapore. The sequencing reactions used the same primers as those used for PCR amplification.

### Sequence alignment

The resulting chromatograms from our sequencing efforts were initially analyzed and edited using Chromas Lite v 2.01 (Technelysium Pty Ltd) to remove any ambiguous bases and refine the sequence data, ensuring high sequence quality. To assess the level of similarity to previously reported sequences and confirm species identification, a Basic Local Alignment Search Tool (BLAST) analysis was conducted against the National Center for Biotechnology Information (NCBI) GenBank database. For multiple sequence alignment, the eight *COI* partial sequences generated from our *Milvus migrans* specimens were processed using BioEdit software [28]. Our newly generated *COI* partial sequences from the eight *Milvus migrans* specimens have been deposited under GenBank accession numbers PQ892169 to PQ892176. An additional 36 *COI* gene sequences of *Milvus migrans* and closely related *Milvus* species were retrieved from the NCBI GenBank and Data Portal – BOLD (The Barcode of Life Data System) systems databases. These publicly available sequences originated from diverse geographic locations, including Japan (n = 13), Nigeria (n = 3), Czechia (n = 5), Germany (n = 5), India (n = 4), Thailand (n = 2), Russia (n = 1), and Northern Mariana Islands (n = 1), along with two additional *M. migrans* sequences from Pakistan (n = 2). In total, 44 *COI* gene sequences were utilized for the comprehensive analyses (Table 1).

**Table 1:** Details of *Milvus* species *COI* gene sequences used in this study, including newly generated and reference sequences from GenBank.

Species Name	Origin	Accession	Reference
*Milvus migrans*	Patisar Lake, Pakistan	PQ892169	This Study
*Milvus migrans*	Taunsa Barrage, Pakistan	PQ892170	This Study
*Milvus migrans*	Taunsa Barrage, Pakistan	PQ892171	This Study
*Milvus migrans*	Taunsa Barrage, Pakistan	PQ892172	This Study
*Milvus migrans*	Chashma Barrage, Pakistan	PQ892173	This Study
*Milvus migrans*	Taunsa Barrage, Pakistan	PQ892174	This Study
*Milvus migrans*	Chashma Barrage, Pakistan	PQ892175	This Study
*Milvus migrans*	Patisar Lake, Pakistan	PQ892176	This Study
*Milvus migrans*	Japan	AB842941	[29]
*Milvus migrans*	Japan	AB842942	[29]
*Milvus migrans*	Japan	AB842938	[29]
*Milvus migrans*	Japan	AB842940	[29]
*Milvus migrans*	Japan	AB842939	[29]
*Milvus migrans*	Japan	AB842937	[29]
*Milvus migrans*	Pakistan	JN801326	[30]
*Milvus migrans*	Japan	DBRCJ058	GenBank Submission
*Milvus migrans*	Japan	DBRCJ059	GenBank Submission
*Milvus migrans*	Japan	DBRCJ060	GenBank Submission
*Milvus migrans*	Nigeria	MH536178	GenBank Submission
*Milvus migrans*	Nigeria	MH536179	GenBank Submission
*Milvus migrans*	Nigeria	JX160002	[31]
*Milvus migrans*	Czechia	KU640398	[32]
*Milvus migrans*	Czechia	KU640403	[32]
*Milvus migrans*	Germany	KU640407	[32]
*Milvus migrans*	India	KP975251	[33]
*Milvus migrans*	India	KP975252	[33]
*Milvus migrans*	India	KP975253	[33]
*Milvus migrans*	Czechia	KU640399	[32]
*Milvus migrans*	Germany	KU640400	[32]
*Milvus migrans*	Czechia	KU640401	[32]
*Milvus migrans*	Germany	KU640402	[32]
*Milvus migrans*	Germany	KU640404	[32]
*Milvus migrans*	Germany	KU640405	[32]
*Milvus migrans*	Czechia	KU640406	[32]
*Milvus migrans govinda*	Thailand	MK932893	GenBank Submission
*Milvus migrans lineatus*	Thailand	MK932894	GenBank Submission
*Milvus migrans*	Russia	MT773184	[27]
*Milvus migrans*	India	OL638139	GenBank Submission
*Milvus migrans*	Northern Mariana Islands	JQ175385	[34]
*Milvus migrans*	Pakistan	JQ175384	[34]
*Milvus migrans*	Japan	YIO1026−24	[29]
*Milvus migrans*	Japan	YIO1042−24	[29]
*Milvus migrans*	Japan	AB843597	[29]
*Milvus migrans*	Japan	YIO708−18	[29]

### Phylogenetic reconstruction

Evolutionary relationships were reconstructed using a robust Bayesian Inference (BI) framework in BEAST v1.10.4 [35] To adhere to methodological parsimony and avoid uncalibrated temporal inferences, a strict molecular clock was enforced with the clock rate fixed at 1.0. Consequently, branch lengths and the associated scale bar represent genetic divergence in units of substitutions per site. The General Time Reversible (GTR + G + I) model was identified as the optimal substitution model via ModelFinder. A Yule speciation prior was utilized to model branching dynamics [36]. The Markov Chain Monte Carlo (MCMC) simulation was executed for 10 million generations, with parameters sampled every 1,000 steps. Convergence was rigorously verified in Tracer v1.7, with all parameters achieving an Effective Sample Size (ESS) > 1,500. A Maximum Clade Credibility (MCC) tree was generated using TreeAnnotator after a 10% burn-in, rooted with the White-tailed Eagle (*Haliaeetus albicilla*), and visualized in FigTree v1.4.4. [37]. Rooting with *H. albicilla* provided a stable reference for interpreting evolutionary relationships within the ingroup [38]. Pairwise genetic distances were obtained using the p-distance model in MEGA 11 [39].

### Statistical analysis

Genetic diversity indices, including haplotype (*H*_*d*_) and nucleotide diversity (π), were computed using DnaSP v6 [40]. To evaluate the spatial partitioning of genetic variance, we performed a hierarchical Analysis of Molecular Variance (AMOVA) in Arlequin v3.5.2.2 [41]. Populations were categorized into three hierarchical groups based on biogeographical proximity and phylogenetic affinity: (1) Punjab Local (newly sequenced samples), (2) Asian Regional (reference sequences from Japan, India, Thailand, and Russia), and (3) Extra Regional (divergent lineages from Europe and Africa). Quantitative levels of genetic differentiation were assessed via pairwise *F*_*ST*_ values with 10,000 permutations. Demographic history was inferred through Tajima’s D and Fu’s *F*_*S*_ neutrality tests. To provide a robust secondary line of evidence for demographic shifts, we generated Mismatch Distributions in DnaSP, comparing observed pairwise differences against expected values under a population growth-decline model. Multivariate analysis, specifically Principal Coordinates Analysis (PCoA), was employed to explore genetic relationships among individuals, and mitochondrial lineage composition was mapped across the study sites.

## Results

### Genetic diversity and haplotype connectivity

The DnaSP analysis identified a total of 15 distinct haplotypes within the mitochondrial *COI* dataset (N = 44). These sequences were partitioned into three hierarchical sets: Punjab_Local (n = 8, newly generated), Asian_Regional (n = 23, conspecific references), and Extra_Regional (n = 13, divergent lineages) (Table 2). The Punjab_Local wetland samples exhibited moderate genetic diversity (*H*_*d*_ = 0.464, *π* = 0.00177), while the Asian Regional group showed slightly lower diversity (*H*_*d*_ = 0.379, *π* = 0.00095). In contrast, the Extra Regional dataset exhibited the highest levels of variation (*H*_*d*_ = 0.987, *π* = 0.00937), reflecting its composition of disparate global lineages.

**Table 2:** Summary of genetic diversity indices for *Milvus migrans COI* gene sequences.

Population/Set	N	S	h	*H*_*d*_	K	π	π (JC)
Set1 (Punjab_Local)	8	3	3	0.464	0.750	0.00177	0.00177
Set2 (Asian_Regional)	23	2	3	0.379	0.403	0.00095	0.00095
Set3 (Extra_Regional)	13	14	12	0.987	3.974	0.00937	0.00945
Total Data Estimates	44	18	15	0.674	2.413	0.00569	0.00574

N: number of sequences; S: number of segregating sites; h: number of haplotypes; *H*_*d*_: haplotype diversity; K: average number of nucleotide differences; π: nucleotide diversity; π (JC): nucleotide diversity with Jukes-Cantor correction.

**Fig 1:**
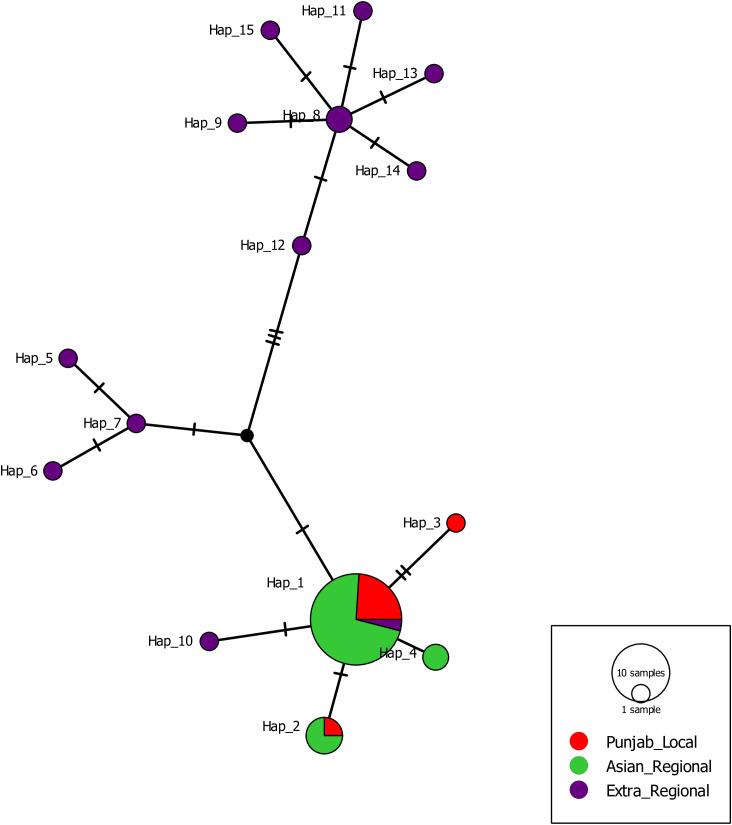
TCS Haplotype network of *Milvus migrans.* Circle sizes correspond to haplotype frequency. Red segments indicate Punjab_Local samples, green indicates Asian_Regional lineages, and purple represents divergent Extra_Regional lineages. The network illustrates the genetic confluence of local and regional Asian haplotypes and their separation from global lineages.

The TCS haplotype network (Fig 1) visually elucidated the complex maternal relationships among these lineages. A dominant, high-frequency central haplotype (Hap_1) served as a phylogenetic hub, encompassing the majority of local samples from Taunsa Barrage, Patisar Lake, and Chashma Barrage, alongside references from Asian_Regional populations (Japan, Pakistan, India, Thailand). Radiating from this hub were several closely related haplotypes differing by 1–2 mutational steps. Distinct clusters representing Extra_Regional lineages (Nigeria, Europe) were significantly more divergent, forming peripheral nodes in the network. The observed maternal connectivity between Punjab and wider Asian populations, contrasted with the isolation of European/African lineages, underscores the Punjab Wetlands as a significant genetic crossroads for Palearctic Black Kite lineages.

### Phylogenetic admixture and lineage relationships

**Fig 2:**
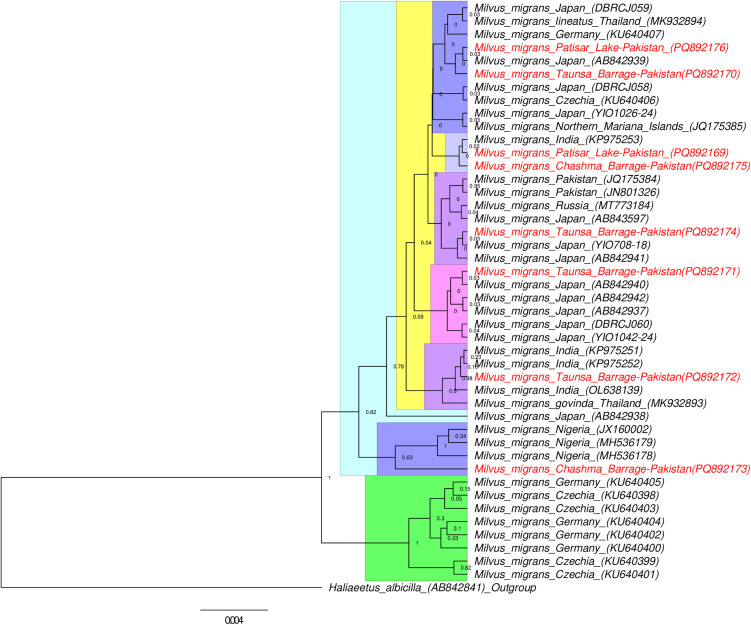
Bayesian Maximum Clade Credibility (MCC) tree of *Milvus migrans* based on mitochondrial *COI* gene sequences. Punjab Wetland sequences are highlighted in red. Values at nodes represent Bayesian posterior probabilities. The tree is rooted with *Haliaeetus albicilla*. Shaded blocks delineate major clades: Green (European lineage), Blue (African/Chashma admixture), and the large Asian complex (Yellow/Pink/Purple). Scale bar represents genetic divergence in substitutions per site.

The Bayesian phylogenetic tree (Fig 2) distinctly revealed a hierarchical structuring of mitochondrial lineages within *Milvus migrans* relative to the *Haliaeetus albicilla* outgroup. The topology highlights the polyphyletic nature of the maternal lineages present within the Punjab local wetlands (indicated in red). The ingroup was broadly divided into three primary clades. The basal clade (Green, posterior probability = 1) predominantly comprised maternal lineages from Europe, specifically Germany (KU640405, KU640404, KU640402, KU640400) and Czechia (KU640398, KU640403, KU640399, KU640401). This clade represents a distinct European *M. migrans* lineage, likely corresponding to *M. m. migrans*. Notably, none of the newly generated samples from the Punjab wetlands clustered within this European mitochondrial lineage. In contrast, the remaining Punjab samples were distributed across the more derived Asian and African lineages. A significant secondary clade (Blue, posterior probability = 0.82) grouped our Chashma Barrage sample (PQ892173) with West African lineages from Nigeria (JX160002, MH536179, MH536178). This intermingling suggests a deeper phylogenetic link between certain Indus Flyway populations and Western Palearctic/Afrotropical lineages. The majority of Punjab samples (Patisar Lake PQ892176, Taunsa Barrage PQ892170, PQ892174) were nested within a large, heterogeneous Asian cluster (Yellow/Pink/Purple backgrounds). These local sequences clustered closely with *M. m. lineatus* and *M. m. govinda* references from Japan, India, Thailand, and Russia. The polyphyletic distribution of the eight newly sequenced samples across these divergent clades provides robust evidence of the Punjab wetlands as a significant crossroads for multiple mitochondrial lineages.

### Genetic distance analysis

The maternal genetic distance analysis, utilizing the p-distance model, provided fine-scale insights into the evolutionary relationships among the 44 *Milvus migrans COI* sequences (detailed in S1 Table). Overall, the evolutionary divergence patterns among the majority of samples were remarkably low. Most regional sequences exhibited high genetic similarity, with pairwise divergences predominantly ranging from 0.00% to 1.00%. This low distance score indicates that these individuals are closely related and represent conspecific maternal lineages. Specifically, newly generated sequences from Patisar Lake, Taunsa Barrage, and Chashma Barrage (with the exception of PQ892173) showed genetic distances between 0.00% and 1.00% when compared with regional references from Japan, India, Thailand, and Russia.

A more pronounced genetic divergence was observed when comparing Indus Flyway populations with extra-regional lineages. Genetic distances between Punjab samples and those from Europe (Czechia and Germany), as well as West Africa (Nigeria), ranged between 1.00% and 2.00%. The European lineage represented by sequence KU640403 exhibited a genetic distance of 2.00% from the Nigerian sequence MH536178. The greatest intraspecific maternal divergence was consistently observed at 2.00%, primarily occurring between Afrotropical and Western Palearctic lineages (S1 Table). This significant partitioning underscores the presence of distinct mitochondrial lineages co-occurring within the *M. migrans* species complex.

### Hierarchical population structure and admixture

**Fig 3:**
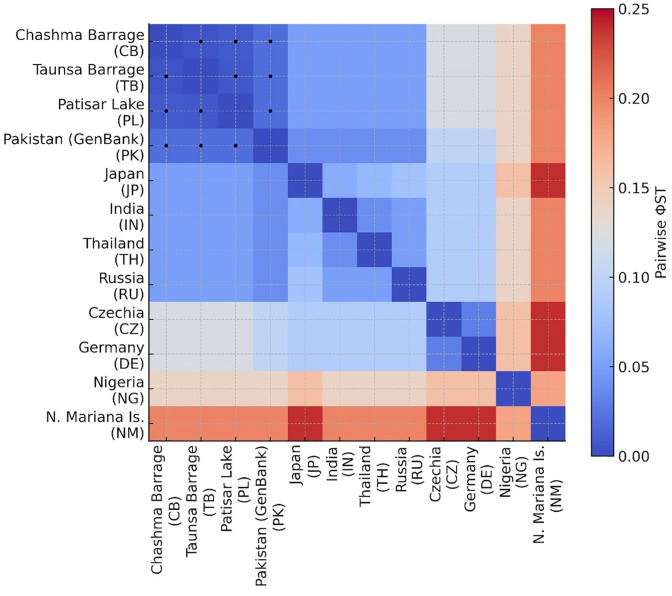
Heatmap of pairwise *F*_*ST*_ values. Genetic differentiation among *M. migrans* populations. Brackets on the axes indicate the hierarchical groups: Punjab_Local, Asian_Regional, and Extra_Regional. Warmer colors indicate higher differentiation; black dots represent non-significant values (P > 0.05).

The hierarchical AMOVA underscored a profound continental-scale genetic structuring. A significant 65.39% of the total genetic variation was partitioned among the three defined groups (*F*_*ST*_ = 0.653, *P* < 0.001), while 34.61% was found within populations (Table 3). Analysis of pairwise *F*_*ST*_ values delineated a clear hierarchy of genetic connectivity. At the regional scale, we found no significant differentiation between the Punjab_Local and Asian_Regional groups (*F*_*ST =*_ −0.046, *P* = 0.536), indicating high gene flow and maternal panmixia across the Asian range. This homogeneity contrasted sharply with the genetic distances observed at broader scales. Differentiation between Punjab and Extra_Regional populations was strikingly high (*F*_*ST =*_ 0.703, *P* < 0.001), confirming the deep divergence of European and African lineages from the Indus Flyway populations. The most profound divisions were observed with geographically isolated lineages from Nigeria and the Northern Mariana Islands (*F*_*ST*_ up to 0.24 in the distance matrix), highlighting the isolating effect of distance on lineage distribution. This pattern of regional connectivity against a backdrop of large-scale structure is summarized in the pairwise heatmap (Fig 3).

**Table 3:** Hierarchical AMOVA results for *Milvus migrans COI* sequences based on defined hierarchical groups.

Source of Variation	*df*	Sum of Squares	Variance Components	% Variation	Fixation Index	*P*-value
Among populations	2	701.744	25.296 Va	65.39%	*F*_*ST*_ = 0.6538*	< 0.001
Within populations	41	549.006	13.390 Vb	34.61%		
Total	43	1250.750	38.687	100%		

Significance of *F*-statistic values was tested using a non-parametric permutation approach described in Excoffier, Smouse [42] with 1,023 permutations: *P < 0.001; *df* degrees of freedom.

### PCoA and lineage composition

**Fig 4:**
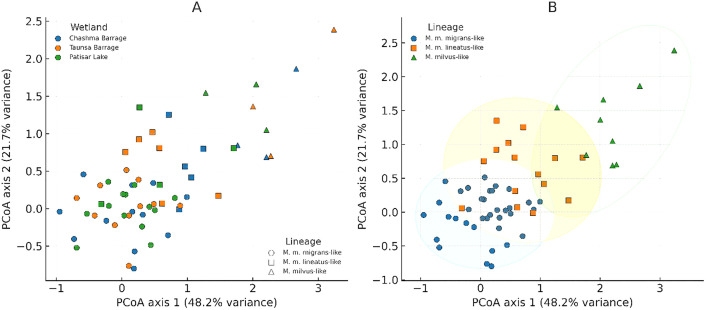
Principal coordinate analysis of *COI* variation in Pakistani *Milvus migrans.* (A) PCoA of pairwise genetic distances among individuals from Chashma Barrage, Taunsa Barrage, and Patisar Lake. Points are colored by wetland and shaped by mitochondrial lineage (*M. m. migrans*-like,*M. m. lineatus*-like, *M. milvus*-like). (B) The same ordination colored by lineage, with 95% confidence ellipses enclosing each lineage cluster. Pakistani individuals largely cluster together, confirming low differentiation among wetlands, but subtle separation of *M. milvus*-like haplotypes suggests a distinct evolutionary background and potential introgression.

Multivariate analysis via PCoA clearly separated the influences of evolutionary lineage from geographical origin. The first two axes accounted for 69.9% of the total variation (48.2% and 21.7%, respectively). Visualization by geographic site showed a complete lack of structure among the three Punjab wetlands, with extensive overlap confirming local panmixia (Fig 4A). However, when categorized by mitochondrial lineage, three distinct genetic clusters were resolved (Fig 4B): an *M. m. migrans* cluster (negative PCoA1), an intermediate *M. m. lineatus* cluster, and a well-separated *M. milvus* cluster (positive PCoA1/PCoA2). The 95% confidence ellipses underscored this pattern, revealing significant differentiation of the *M. milvus* lineage and substantial overlap between *migrans* and *lineatus*, supporting a model of admixture between these two subspecies.

**Fig 5:**
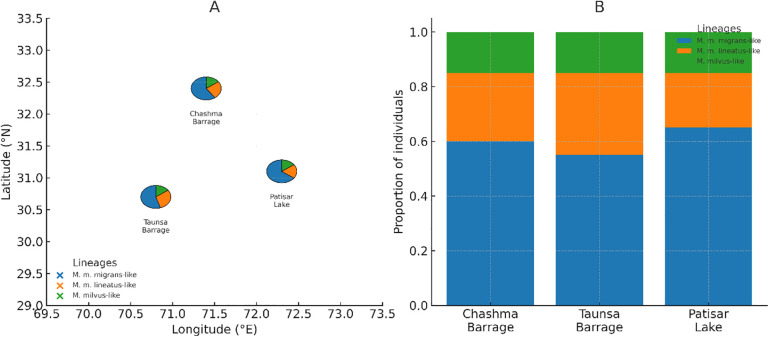
Subspecies lineage composition of *Milvus migrans* in Punjab wetlands. (A) Schematic map of the three sampling wetlands in Punjab, Pakistan (Chashma Barrage, Taunsa Barrage, Patisar Lake) with pie charts showing the proportion of mitochondrial lineages (*M. m. migrans*-like, *M. m. lineatus*-like, and *M. milvus*-like) at each site. (B) Stacked barplot summarizing lineage frequencies for each wetland. The coexistence of *migrans*- and *lineatus*-like haplotypes, together with a distinct *M. milvus*-like lineage, illustrates subspecies admixture and supports potential hybridization along the Indus flyway.

The assignment of *COI* haplotypes to subspecies lineages demonstrated that the Punjab wetland populations constitute a admixed mosaic, with all three mitochondrial lineages *M. m. migrans*, *M. m. lineatus*, and *M. milvus* co-occurring at each site (Fig 5). The *M. m. migrans* lineage was dominant, representing roughly 67% of individuals, followed by *M. m. lineatus*, while the *M. milvus* lineage was consistently present at lower frequencies. Critically, the geographic distribution of these lineages was remarkably homogeneous across the study region. The pie chart visualization (Fig 5A) shows only negligible variations in lineage proportions among Chashma Barrage, Taunsa Barrage, and Patisar Lake. This is corroborated by the stacked bar plot (Fig 5B), leading to the conclusion that this triple-lineage admixture is a region-wide phenomenon within the Indus Flyway, rather than a site-specific occurrence.

### Demographic inference and neutrality

**Fig 6:**
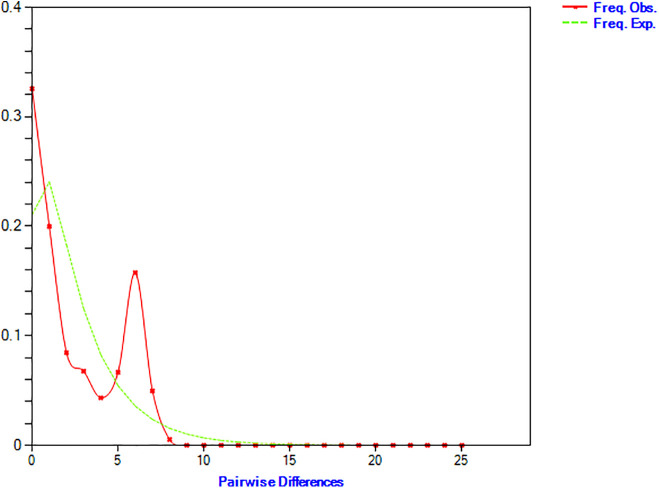
Mismatch distribution of *Milvus migrans COI* sequences. The observed frequency of pairwise differences (red line) compared to the expected population growth-decline model (dashed green line). The multimodal pattern shows the intersection of multiple divergent genetic lineages within the dataset.

Demographic analyses for the Punjab_Local population yielded a significantly negative Tajima’s D value (−1.701, *P* = 0.013), which is traditionally consistent with a recent demographic expansion or the signature of purifying selection acting on the mitochondrial genome. However, the Mismatch Distribution for the overall dataset (Fig 6) exhibited a distinctly multimodal pattern, diverging from the unimodal bell curve expected under a simple population expansion. This complexity, supported by a Harpending’s Raggedness index (*r* = 0.0524) and R_2_ statistic (0.0604), showed that the observed genetic signatures likely reflect the confluence of multiple, divergent lineages with independent demographic histories (the “crossroads” effect) rather than a single, cohesive expansion event.

## Discussion

This research aimed to provide the first integrative genetic assessment of Black Kite populations in Punjab wetlands (Chashma Barrage, Taunsa Barrage and Patisar Lake), clarifying their evolutionary lineages and potential interactions with other *Milvus* taxa. Our analysis identified substantial mitochondrial variability across both regional and global scales, characterized by a moderate degree of haplotype diversity and significant negative neutrality statistics. While the observed negative Tajima’s D in local populations is consistent with demographic expansion following historical bottlenecks [43,44]. We acknowledge that mitochondrial *COI* data are intrinsically susceptible to purifying selection and selective sweeps, which can manifest as similar neutrality signatures. This interpretation is further nuanced by the distinctly multimodal nature of our mismatch distribution; rather than reflecting a singular, cohesive expansion event, this pattern likely denotes a confluence of disparate lineages (the ‘crossroads’ effect). Consequently, we interpret these findings as reflective of complex maternal evolutionary dynamics, the definitive resolution of which remains contingent upon the integration of multi-locus nuclear data to distinguish between regional demographic growth and natural selection on the mitochondrial genome.

At the local scale, the absence of significant genetic differentiation among the three Punjab wetlands suggests a highly connected and panmictic population. This regional homogeneity is likely facilitated by the Black Kite inherent mobility [45,46], a pattern consistent with other wide-ranging raptors where extensive dispersal minimizes regional divergence [47]. However, this local panmixia starkly contrasts with the robust genetic structuring observed at broader, continental scales. Our study results revealed considerable barriers to gene flow beyond regional boundaries. This strong partitioning of genetic variation suggests that while populations within a connected landscape like the Punjab wetlands may interbreed freely, larger geographical distances and barriers effectively limit gene exchange between continental populations.

The phylogenetic reconstruction underscores the Punjab region role as a critical biogeographical hub. The most striking finding is the polyphyletic distribution of the local maternal lineages; rather than forming a single, isolated population, the Punjab samples are dispersed among global clades. Specifically, the nesting of the majority of local sequences within the Asian lineage cluster confirms a strong genetic affinity with eastern Palearctic populations. However, the consistent grouping of the Chashma Barrage sample (PQ892173) with divergent lineages from the Afrotropical/Western Palearctic region illustrates a significant aspect of mitochondrial diversity that has been previously overlooked in South Asia. This aligns with previous studies that have identified overlapping ranges and hybridization between *M. m. migrans* and *M. m. lineatus*, particularly in areas like Central Asia and the Himalayas [2,10]. The challenges in distinguishing these subspecies genetically, especially in regions of overlap where migrants and residents co-occur, have been noted in prior research [7,32]. The genetic distance between *M. migrans* and *M. milvus* is generally small, reflecting their divergence during the Pleistocene, but they are typically considered reproductively isolated species, albeit with incomplete isolation evident through occasional hybridization [32,48]. Documented hybridization between *M. milvus* and *M. migrans* in Central Europe [49–51], confirms that interbreeding occurs among these taxa, albeit infrequently in raptors [52].

In our study, this intermingling of Pakistani samples with distinct mitochondrial lineages suggests that the Indus Flyway serves as a zone of secondary contact or a refugial crossroads for divergent migratory routes. The 0.00% to 2.0% genetic distances observed are characteristic of intraspecific lineage admixture rather than definitive speciation. These results provide a foundational mitochondrial framework that necessitates future validation with multilocus nuclear markers to resolve the biparental dynamics of hybridization and introgression at this ecological intersection [14].

Our study contributes significantly to filling a critical knowledge gap in the genetic characterization of *M. migrans* in South Asia. While previous phylogenetic studies on Black Kites were also limited by sample size, geographic representation and utilized published nucleotide sequences [2,10,32,48,53–55]. Our study highlights the region as a vital crossroads for *Milvus* lineages, underscoring the necessity for conservation strategies that account for genetic connectivity and potential admixture. Future research should prioritize expanded geographical sampling across Pakistan and incorporate multiple nuclear markers to resolve the evolutionary history of *Milvus* species in this critical part of their range.

## Conclusion

Our comprehensive genetic analysis of *M. migrans* populations in the Punjab wetlands has revealed notable genetic variability, identifying 15 distinct haplotypes. This analysis underscores the complexity and diversity of maternal lineages within the species. The phylogenetic clustering of our Punjab Wetland isolates among diverse global lineages suggests that this region serves as a vital confluence for *Milvus migrans* populations. Rather than confirming cryptic speciation, our data point toward a shared yet intricate evolutionary history shaped by high connectivity and the convergence of distinct Palearctic lineages.

## Supporting information

S1 Table Pairwise genetic distances (*p*-distance) among *Milvus migrans* mitochondrial *COI* gene sequences. The lower triangular part displays genetic distances in percentages; the upper triangular part shows the associated standard errors.
(PDF)

S1 Fig Original uncropped and unadjusted gel electrophoresis images of mitochondrial *COI* gene PCR products. Electrophoretic separation was conducted on a 1.5% agarose gel and visualized via UV transillumination. Lane M denotes a 100 bp DNA ladder (ranging from 100 bp to 1500 bp). The observed bands at approximately 700 bp confirm the successful amplification of the target *COI* gene segment for the Punjab wetland samples (labeled P1–P3 and Q2–Q6).
(PDF)

## References

[1] Meyburg B, Christie D, Kirwan G, Marks J. Handbook of the birds of the world alive. CHOICE: Current Reviews for Academic Libraries; 2018.

[2] Karyakin I. Problem of Identification of Eurasian Subspecies of the Black Kite and Records of the Pariah Kite in Southern Siberia, Russia. Raptors Conserv. 2017;(34):49–67. doi: 10.19074/1814-8654-2017-34-49-67

[3] Roberts TJ. The birds of Pakistan. Oxford University Press; 1991.

[4] Hong S-Y, Lin H-S, Walther BA, Shie J-E, Sun Y-H. Recent Avian Poisonings Suggest a Secondary Poisoning Crisis of Black Kites During the 1980s in Taiwan. J Raptor Res. 2018;52(3):326. doi: 10.3356/jrr-17-40.1

[5] Carter I. The red kite reintroduction: 30 years on. British Birds. 2019;112(8):422–6.

[6] BirdLife International. Red Kite *Milvus milvus*. IUCN Red List Threat Species. 2019.

[7] Andreyenkova NG, Hong S-Y, Lin H-S, Iwami Y, Kirillin RA, Literák I, et al. Genetic relationships of populations of the Black Kite Milvus migrans (Accipitriformes: Accipitridae) in the east of its range in Asia and Australia. Zootaxa. 2024;5523(1):83–99. doi: 10.11646/zootaxa.5523.1.5 39645951

[8] BirdLife International. Species factsheet: Milvus migrans. 2020 [09 August 2020]. Available from: www.birdlife.org

[9] Avise JC, Bowen BW, Ayala FJ. In the light of evolution X: Comparative phylogeography. Proc Natl Acad Sci U S A. 2016;113(29):7957–61. doi: 10.1073/pnas.1604338113 27432955 PMC4961136

[10] Andreyenkova NG, Karyakin IV, Starikov IJ, Sauer‐Gürth H, Literák I, Andreyenkov OV, et al. Phylogeography and demographic history of the black kite *Milvus migrans*, a widespread raptor in Eurasia, Australia and Africa. Journal of Avian Biology. 2021;52(10). doi: 10.1111/jav.02822

[11] Andreyenkova NG, Starikov I, Wink M, Karyakin I, Andreyenkov O, Zhimulev I. The problems of genetic support of dividing the black kite (*Milvus migrans*) into subspecies. Вавиловский журнал генетики и селекции. 2019;23(2):226–31. doi: https://doi.org/10.18699/vj19.486

[12] Skyrpan M, Panter C, Nachtigall W, Riols R, Systad G, Škrábal J, et al. Kites *Milvus migrans lineatus* (*Milvus migrans migrans/lineatus*) are spreading west across Europe. J Ornithol. 2021;162(2):317–23. doi: 10.1007/s10336-020-01832-2

[13] Trivedi S, Rehman H, Saggu S, Panneerselvam C, Ghosh SK. DNA barcoding and molecular phylogeny. Springer; 2020.

[14] Marín-Villa J, López-Herrera A, Gómez-Ruiz DA, Restrepo-Rodas DC, Sánchez-Rodríguez G, Úsuga-Monroy C. Mitochondrial markers (cytochrome c oxidase subunit I and 16S ribosomal RNA) as supporting biomarkers for wild bird identification. Vet World. 2025;18(5):1389–99. doi: 10.14202/vetworld.2025.1389-1399 40584119 PMC12205242

[15] Hussain J, Afzal G, Haider MZ, Qadeer I, Perveen S, Ahmad HI, et al. Genetic diversity and phylogenetic relationships of *Calotes* and *Uromastyx* in the Cholistan Desert, Pakistan, based on *COI* gene analysis. PLoS One. 2025;20(6):e0324053. doi: 10.1371/journal.pone.0324053 40526730 PMC12173366

[16] Buthasane W, Tangphatsornruang S, Jenjaroenpun P, Wongsurawat T, Sanannu S, Shotelersuk V. Complete mitogenome of the critically endangered Asian king vulture (*Sarcogypscalvus*) (*Aves*, *Accipitriformes*, *Accipitridae*): evolutionary insights and comparative analysis. ZooKeys. 2025;1234:47–65. doi: https://doi.org/10.3897/zookeys.1234.138722 40248455 10.3897/zookeys.1234.138722 PMC12000817

[17] Sheikh K, Kashif N. Strategic role of Pakistan wetland resources: prospects for an effective migratory waterbird conservation network. Waterbirds around the world. 2006. p. 292–3.

[18] Umar M, Hussain M, Murtaza G, Shaheen FA, Zafar F. Ecological Concerns of Migratory Birds in Pakistan: A Review. Punjab Univ J Zool. 2018;33(1). doi: 10.17582/pujz/2018.33.1.69.76

[19] Galbraith CA. A review of migratory bird flyways and priorities for management. UNEP/CMS Secretariat; 2014.

[20] Dawn T. Number of birds migrating from Siberia to Pakistan declines. 2016.

[21] Haider MZ, Ahmed S, Sial N, Afzal G, Riaz A, Asif AR, et al. Avian Diversity and Abundance of Taunsa Barrage Ramsar Site in Punjab, Pakistan. J Zool Systemat Evolution Res. 2022;2022:1–14. doi: 10.1155/2022/4736195

[22] Rasool G, Aihetasham A, Ali Z, Ahmad R. Avian Richness, Assemblages and Migration Connectivity of Geese Species with Habitat Suitability in Wetlands of the Punjab, Pakistan. PJZ. 2024;56(5). doi: 10.17582/journal.pjz/20230724085011

[23] Juhant MA, Bildstein KL. Raptor migration across and around the Himalayas. Bird migration across the Himalayas: wetland functioning amidst mountains and glaciers. 2017. p. 98–116.

[24] Batool A, Parveen A, Nawaz M, Razzaq D, Mukhtar M, Mustafavi N. Wetlands of Plains of Pakistan. Wetlands of Tropical and Subtropical Asia and Africa: Biodiversity, Livelihoods and Conservation. 2025. p. 67–83.

[25] Berger DD, Mueller HC. The Bal-Chatri: A Trap for the Birds of Prey. Bird-Banding. 1959;30(1):18. doi: 10.2307/4510726

[26] Chevrinais M, Larivière J, Dumoulin L-A, Therien A, Cortial G, Lepage C, et al. Optimized QIAGEN DNeasy blood & tissue kit protocol for environmental DNA extraction. 2023. doi: 10.17504/protocols.io.8epv5xn66g1b/v1

[27] Silaeva OL, Kholodova MV, Sviridova TV, Bukreev SA, Varaksin AN. Research on Aircraft Collisions with Birds according to Identification Examinations in 2002–2019. Biol Bull Russ Acad Sci. 2020;47(6):624–32. doi: 10.1134/s1062359020060126

[28] Hall TA. BioEdit: a user-friendly biological sequence alignment editor and analysis program for Windows 95/98/NT. Nucleic acids symposium series. Oxford; 1999.

[29] Saitoh T, Sugita N, Someya S, Iwami Y, Kobayashi S, Kamigaichi H, et al. DNA barcoding reveals 24 distinct lineages as cryptic bird species candidates in and around the Japanese Archipelago. Mol Ecol Resour. 2015;15(1):177–86. doi: 10.1111/1755-0998.12282 24835119

[30] Stoeckle M. All birds barcoding initiative. unpublished data. 2011.

[31] Echi P, Suresh L, George S, Ratheesh R, Ezeonu I, Ejere V, et al. Molecular resolution of some West African birds using DNA barcoding. Environ Conserv J. 2015;16(1&2):87–92. doi: 10.36953/ECJ.2015.161214

[32] Heneberg P, Dolinay M, Matušík H, Pfeiffer T, Nachtigall W, Bizos J, et al. Conservation of the Red Kite Milvus milvus (Aves: Accipitriformes) Is Not Affected by the Establishment of a Broad Hybrid Zone with the Black Kite Milvus migrans migrans in Central Europe. PLoS One. 2016;11(7):e0159202. doi: 10.1371/journal.pone.0159202 27463515 PMC4962980

[33] Gaikwad SS, Munot H, Shouche YS. Utility of DNA barcoding for identification of bird-strike samples from India. Curr Sci. 2016;110(1):25–8.

[34] Schindel DE, Stoeckle MY, Milensky C, Trizna M, Schmidt B, Gebhard C. Project description: DNA barcodes of bird species in the national museum of natural history, smithsonian institution, USA. ZooKeys. 2011;152:87. doi: https://doi.org/10.3897/zookeys.152.2473 10.3897/zookeys.152.2473 PMC3234427 22287908

[35] Suchard MA, Lemey P, Baele G, Ayres DL, Drummond AJ, Rambaut A. Bayesian phylogenetic and phylodynamic data integration using BEAST 1.10. Virus Evol. 2018;4(1):vey016. doi: 10.1093/ve/vey016 29942656 PMC6007674

[36] Gernhard T. The conditioned reconstructed process. J Theor Biol. 2008;253(4):769–78. doi: 10.1016/j.jtbi.2008.04.005 18538793

[37] Rambaut A. FigTree v1. 4.2, a graphical viewer of phylogenetic trees. 2014. Java https://githubcom/rambaut/figtree.2018

[38] Hansen CCR. White-tailed eagles in time and space.: University of Iceland, School of Engineering and Natural Sciences, Faculty of Life and Environmental Sciences. 2021.

[39] Tamura K, Stecher G, Kumar S. MEGA11: Molecular Evolutionary Genetics Analysis Version 11. Mol Biol Evol. 2021;38(7):3022–7. doi: 10.1093/molbev/msab120 33892491 PMC8233496

[40] Rozas J, Ferrer-Mata A, Sánchez-DelBarrio JC, Guirao-Rico S, Librado P, Ramos-Onsins SE, et al. DnaSP 6: DNA Sequence Polymorphism Analysis of Large Data Sets. Mol Biol Evol. 2017;34(12):3299–302. doi: 10.1093/molbev/msx248 29029172

[41] Excoffier L, Lischer HEL. Arlequin suite ver 3.5: a new series of programs to perform population genetics analyses under Linux and Windows. Mol Ecol Resour. 2010;10(3):564–7. doi: 10.1111/j.1755-0998.2010.02847.x 21565059

[42] Excoffier L, Smouse PE, Quattro JM. Analysis of molecular variance inferred from metric distances among DNA haplotypes: application to human mitochondrial DNA restriction data. Genetics. 1992;131(2):479–91. doi: 10.1093/genetics/131.2.479 1644282 PMC1205020

[43] Burke MB. Population genetics of the bearded vulture (Doctoral dissertation). Pietermaritzburg: University of KwaZulu-Natal; 2018.

[44] Montaño‐Centellas F, Baiser B, McGrew A, Trotta L, Li D. Global patterns and drivers of raptor phylogenetic and functional diversity. Glob Ecol Biogeogr. 2022;32(2):281–94. doi: 10.1111/geb.13619

[45] Rashid GM, Butt A, Qadir A, Ali MH. Exploring black kite (Milvus migrans) dynamics: Seasonal abundance and habitat preferences in an urban gradient. J Asia-Pac Biodivers. 2025;18(1):101–7. doi: 10.1016/j.japb.2024.07.005

[46] Haque MA, Ahammed R, Islam MA, Khan MNH, Rayan SA, Khan MMH, et al. Population status and feeding behavior of black kite (*Milvus migrans*) in Dhaka city, Bangladesh. Jahangirnagar Univ J Biol Sci. 2020;9(1–2):35–48. doi: https://doi.org/10.3329/jujbs.v9i1-2.53705

[47] Hailer F, Helander B, Folkestad AO, Ganusevich SA, Garstad S, Hauff P, et al. Phylogeography of the white‐tailed eagle, a generalist with large dispersal capacity. J Biogeogr. 2007;34(7):1193–206. doi: 10.1111/j.1365-2699.2007.01697.x

[48] Simakov M, Korepov M, Kuzmin A, Titov S. Genetic diversity of mtDNA in the Volga black kite population (*Milvus migrans* Boddaert, 1783). Russ J Ecosyst Ecol. 2021;6. doi: https://doi.org/10.21685/2500-0578-2021-1-5

[49] Ovčiariková S, Škrábal J, Matušík H, Makoň K, Mráz J, Arkumarev V, et al. Natal dispersal in Black Kites *Milvus migrans* migrans in Europe. J Ornithol. 2020;161(4):935–51. doi: 10.1007/s10336-020-01780-x

[50] Literák I, Kyseláková CM, Dostál M, Karlsson C, Škrábal J, Skyrpan M, et al. Evidence of genetic determination of annual movement strategies in medium-sized raptors. Sci Rep. 2025;15(1):3159. doi: 10.1038/s41598-025-86414-z 39856092 PMC11760365

[51] Literák I, Balla M, Vyhnal S, Škrábal J, Peške L, Chrašč P, et al. Natal dispersal of black kites from Slovakia. Biologia. 2019;75(4):591–8. doi: 10.2478/s11756-019-00323-x

[52] Väli Ü. Autumn Migration of a Juvenile Hybrid of Red and Black Kite (*Milvus milvus × M. migrans*). Ardea. 2024;112(1). doi: 10.5253/arde.2023.a11

[53] Nagai K, Tokita K i. Analysis of genetic structure and genetic diversity in Japanese Black Kite population using mtDNA. Zool Sci. 2022;39(4):330–5. doi: 10.2108/zs210121 35960032

[54] Andreyenkova N, Lin HS, Karyakin I. Taiwanese Black Kite: Does the Subspecies formosanus Exist?. Raptors Conserv. 2023;46:34–45. doi: https://doi.org/10.19074/1814-8654-2023-46-34-45

[55] Mindell DP, Fuchs J, Johnson JA. Phylogeny, taxonomy, and geographic diversity of diurnal raptors: Falconiformes, Accipitriformes, and Cathartiformes. Birds of prey: biology and conservation in the XXI century. Springer; 2018. p. 3–32. 10.1007/978-3-319-73745-4_1

