# Supplementary material for: Phylogeography of the Black Kite (Milvus migrans) in Punjab Wetlands: Assessing genetic connectivity and lineage admixture at a migratory crossroads
Source: PLoS One. 2026 Jun 29;21(6):e0351642. doi: 10.1371/journal.pone.0351642 (PMC13313336; doi:10.1371/journal.pone.0351642)

S1 Raw Images. Original uncropped and unadjusted gel electrophoresis images of mitochondrial *COI* gene PCR products.

The provided files contain the raw, unedited captured data underlying the molecular identification of *Milvus migrans*. Electrophoretic separation was conducted on a 1.5% agarose gel and visualized via UV transillumination. Lane M denotes a 100 bp DNA ladder (ranging from 100 bp to 1500 bp). The observed bands at approximately 700 bp confirm the successful amplification of the target *COI* gene segment for the Punjab wetland samples (labeled P1–P3 and Q2–Q6).

Labeled Image

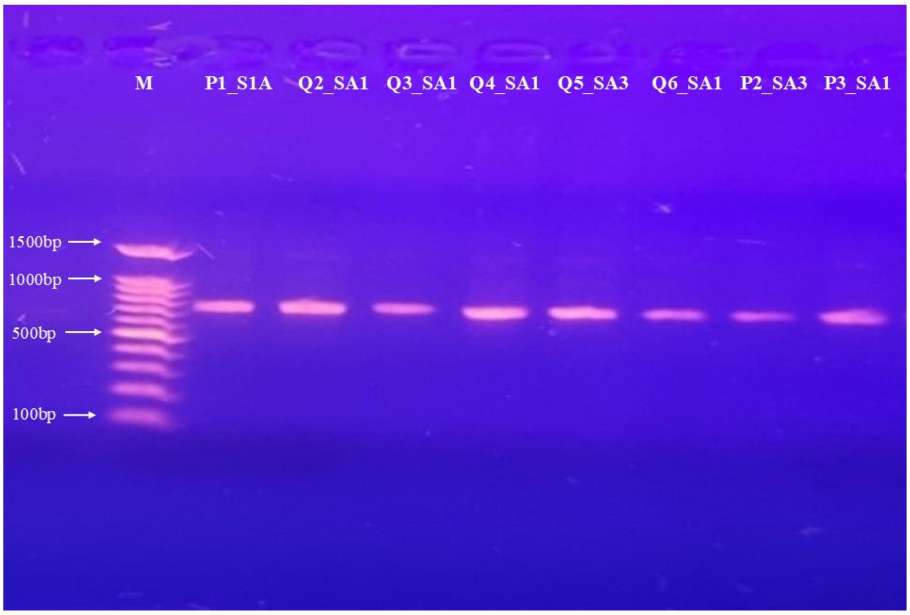

Original Image

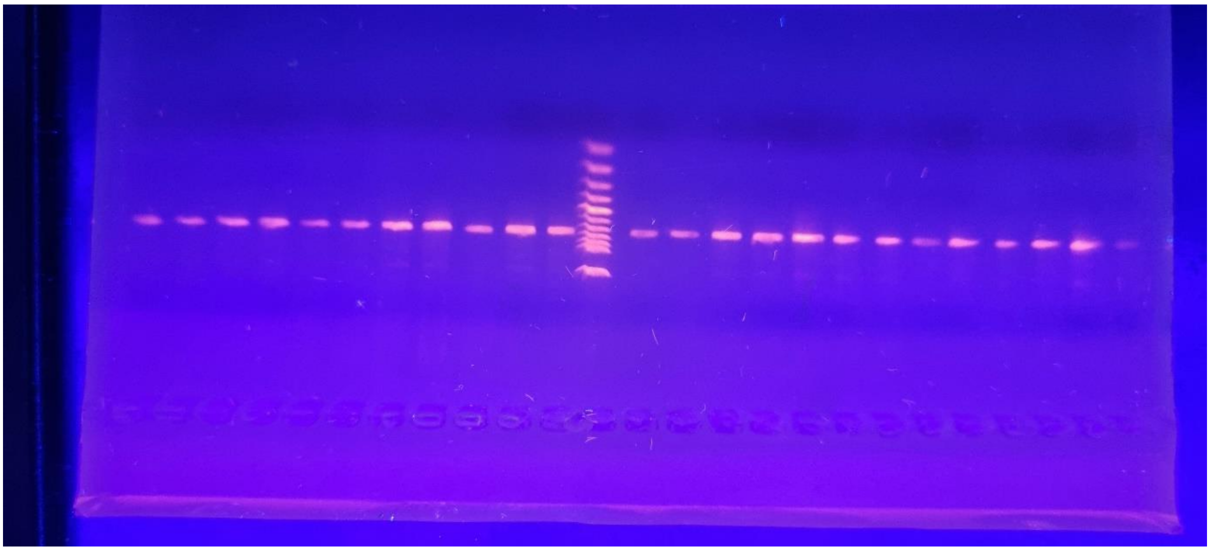

Supplement: S1 Fig — Electrophoretic separation was conducted on a 1.5% agarose gel and visualized via UV transillumination. Lane M denotes a 100 bp DNA ladder (ranging from 100 bp to 1500 bp). The observed bands at approximately 700 bp confirm the successful amplification of the target COI gene segment for the Punjab wetland samples (labeled P1–P3 and Q2–Q6). (PDF) [file pone.0351642.s002.pdf]
